# 

**Published:** 1897-10

**Authors:** 


### Communicated.

### NORTHERN ILLINOIS DENTAL SOCIETY.

The tenth annual meeting of the Northern Illinois Dental Society will be held at Rockford, October 20 and 21, 1897. A great program is in the course of preparation, and the profession generally is invited to attend.

LOUIS OTTOFY,
Chairman Executive Committee.
J. W. CORMANY,
Secretary.

### To Readers and Correspondents.

Communications intended for publication, and exchange journals and papers, should be addressed to the Editor of The American Journal of Dental Science, No. 845 N. Eutaw St. Baltimore, Md. All letters relating to the business of the Journal, or containing cash remittances, to be directed to Snowden & Cowman Mfg. Co., 9 W. Fayette Street, Baltimore, Md. Articles for publication should be received by the Editor at least two weeks previous to the first of the month on which the number in which it is designed for them to appear is issued.

☞ The American Journal of Dental Science will contain fifty pages of reading matter in each number, making a volume of about Six Hundred pages,

EXCLUSIVE OF ADVERTISEMENTS.

